# Biology of Platelet Purinergic Receptors and Implications for Platelet Heterogeneity

**DOI:** 10.3389/fphar.2018.00037

**Published:** 2018-01-30

**Authors:** Milka Koupenova, Katya Ravid

**Affiliations:** ^1^Department of Medicine, Division of Cardiovascular Medicine, University of Massachusetts Medical School, Worcester, MA, United States; ^2^Departments of Medicine and Biochemistry and Whitaker Cardiovascular Institute, Boston University School of Medicine, Boston, MA, United States

**Keywords:** platelets, purinergic receptors, ATP, ADP, adenosine

## Abstract

Platelets are small anucleated cells present only in mammals. Platelets mediate intravascular hemostatic balance, prevent interstitial bleeding, and have a major role in thrombosis. Activation of platelet purinergic receptors is instrumental in initiation of hemostasis and formation of the hemostatic plug, although this activation process becomes problematic in pathological settings of thrombosis. This review briefly outlines the roles and function of currently known platelet purinergic receptors (P1 and P2) in the setting of hemostasis and thrombosis. Additionally, we discuss recent novel studies on purinergic receptor distribution according to heterogeneous platelet size, and the possible implication of this distribution on hemostatic function.

## Introduction

Adenine nucleosides and nucleotides accumulate in the circulation under various pathological conditions and can initiate and mediate the response to cell damage, hypoxia, and inflammation (Koupenova and Ravid, 2013). Additionally, adenosine diphosphate (ADP) and adenosine triphosphate (ATP) are released from dense-granules during platelet activation and propagate platelet–platelet interactions, ultimately leading to three-dimensional plug formation that seals endothelial damage (McNicol and Israels, 1999; Koupenova and Ravid, 2013; Koupenova et al., 2017). Adenosine, in turn, is not known to be present in platelets but is generated in the extracellular space from ATP and ADP by two ectonucleotidases, CD39 (ENTPD1) and CD73 (NT5E) (reviewed in Koupenova and Ravid, 2013), that are present on endothelial and platelet surfaces (Koziak et al., 1999; Castilhos et al., 2016).

Two major classes of receptors mediate the physiological effect of adenosine and adenosine phosphates. These are the P1 and P2 purinergic receptors, classified based on their preference for adenosine (P1) or adenosine phosphates (P2). The P1 purinergic receptors include four G-protein-coupled receptors, two that activate adenylate cyclase [Adora2A (A2aAR) and Adora2b (A2bAR)] and generate cyclic AMP (cAMP), and two that inhibit adenylate cyclase [AdoraA1 (A1AR) and AdoraA3 (A3AR)] and decrease cAMP. The P2 purinergic receptor group is subdivided further into two groups that, in platelets, are represented by receptors activated by ATP (P2X1) and receptors activated by ADP (P2Y1 and P2Y12). P2Y1 and P2Y12 are G-protein-coupled receptors, while P2X1 is a ligand-gated ion channel receptor (Jacobson et al., 2006; Cattaneo, 2007). Adenosine and adenosine phosphates play a central role in regulating platelet behavior during hemostasis and thrombosis, as ADP induces platelet activation, while adenosine, for the most part, inhibits it. ATP, in turn, can initiate platelet activation through the P2X1 receptor and inhibit ADP-mediated activation when acting as an antagonist on the P2Y receptors (Cattaneo, 2007). The potential explanation as to why platelets have both ATP and ADP receptors is that all of the purinergic receptors are necessary for establishing a balanced regulation of hemostasis or thrombus formation, depending on the intensity of vessel injury. Nucleotides released from damaged cells may occupy platelet P2 receptors and initiate shape change (via P2X receptors) and aggregation (via P2Y12 and P2Y1 receptor). The level of ADP-mediated P2Y12 or P2Y1 receptor activation can be controlled by ATP. Conversion of ATP and ADP (by CD39/CD73) to adenosine provides a second level of regulation that controls propagation of the hemostatic plaque by inhibiting activation through the A2 ARs. Deeper damage of the vessel leads to an increase in platelet aggregation response (collagen- or tissue factor-mediated) in order to prevent leakage into the interstitial tissue. This simplified explanation, however, is complicated by the fact that platelets are a heterogeneous population (as discussed in the last section of this review) and there is differential expression of distinct purinergic receptors based on size and function. In this review, we will focus on the role of purinergic receptors in platelet function and their distribution according to platelet heterogeneity.

## Purinergic Receptors in Hemostasis

Recent *in vivo* studies have further clarified the complex interactions and agonist distribution in the formation of three-dimensional platelet arrangement during hemostasis. According to this newly elucidated model, the hemostatic plug is composed of a core and an outer shell through which platelets are differentially activated. The stringent plug architecture consists of a platelet activation gradient with the most activated platelets in the core of the clot, surrounded by less activated platelets in the outer shell region. Fibrin deposition is localized distinctly at the base of the core in the extravascular space before hemostasis is achieved (Stalker et al., 2013; Tomaiuolo et al., 2017). The inner core of the hemostatic plug is packed tightly with degranulated platelets that are P-selectin positive. The outer shell is composed of loosely packed platelets that do not express P-selectin, and there is little to no fibrin present.

Although stable, the outer shell is porous and permeable to plasma solutes. Consistent with the platelet activation distribution gradient, there is a distinct distribution of platelet agonists throughout the hemostatic plug. The core of the plug contains a high concentration of thrombin (factor IIa) and, as the plug becomes more porous, a gradient of ADP and thromboxane A2 (TxA2) develops (Stalker et al., 2013; Tomaiuolo et al., 2017). The porous outer shell of the thrombus allows for recruitment of leukocytes necessary for injury repair or pathogen elimination. An increase in thrombin leads to PAR4 cleavage, consequently leading to leukocyte recruitment and migration to the damaged endothelium (Kaplan et al., 2015). Leukocyte recruitment, in turn, is limited by binding of thrombin to platelet GP1bα that can reduce platelet activation. Additionally, fibrin deposition in the thrombus physically inhibits leukocyte migration (Kaplan et al., 2015). The distinct distribution of P-selectin expressing platelets in the core vs. P-selectin-negative platelets in the shell suggests a possibility for a specific distribution of different platelet subpopulations throughout the hemostatic plug, according to their function in the interaction with either damaged endothelium or circulating leukocytes.

## Platelets and P2 Receptors

### ATP Receptors in Platelets

The P2X1 receptor is a ligand-gated ion channel receptor (Sun et al., 1998) that is activated by ATP and inhibited by ADP. Binding of ATP to the P2X1 receptor leads to calcium influx into platelets (Rolf et al., 2001; Mahaut-Smith, 2012) which consequently results in a transient change of platelet shape, platelet degranulation, pseudopodia formation, and platelet activation (Rolf et al., 2001; Toth-Zsamboki et al., 2003; Mahaut-Smith, 2012). P2X1 receptor activation by ATP alone does not mediate platelet aggregation; however, it can amplify ADP-mediated aggregation through the platelet-P2Y1 receptor (Jones et al., 2014). Furthermore, during early stages of vessel damage, in the presence of a low concentration of collagen, ATP contributes to increased aggregation through the P2X1 receptor (Oury et al., 2001). Similarly, P2X1 receptors can amplify thrombin-mediated platelet aggregation through protease-activated receptor 1 (PAR1) at low levels of thrombin (Erhardt et al., 2006). Importantly, in the presence of collagen or pathogenic stimuli, endothelial inhibitors (such as prostacyclins) are unable to completely inhibit calcium-mediated platelet aggregation partially due to activation of P2X1 receptor by ATP (Fung et al., 2012).

Intracellularly, P2X1 activation leads to MAPK/ERK2 pathway signaling that contributes to myosin light chain (MLC) phosphorylation and propagation of collagen-mediated platelet secretion (Toth-Zsamboki et al., 2003). During high shear stress, ATP-activated P2X1 also contributes to platelet-induced aggregation by MLC-mediated cytoskeletal rearrangements (Oury et al., 2004). P2X1 activation by ATP can also contribute to platelet secretion of TxA2 and enhance TxA2-mediated platelet aggregation (Huang et al., 2014). Additionally, in cases of a co-stimulatory role with P2Y1 signaling, P2X1 increases the influx of calcium and amplifies the consequent calcium signaling through P2Y1 and other G_αq_-coupled platelet receptors (Jones et al., 2014). Therefore, at sites of vascular injury, intensity of the platelet response can be regulated by the availability of various forms of adenosine phosphates.

Murine platelets lacking the P2X1 receptor exhibit decreased collagen-induced aggregation and adhesion (Hechler et al., 2003a). Furthermore, these platelets show diminished thrombus growth on collagen-coated slides, particularly at higher shear stress (Hechler et al., 2003a). Overall, P2X1 activation seems to be important at high shear stress and low agonist concentration, suggesting that ATP contributes to platelet aggregation at the initial stages of platelet attachment to damaged endothelium, particularly in the arteries.

### ADP Receptors in Platelets

P2Y12 and P2Y1 receptors are G-protein-coupled receptors activated by ADP and inhibited by ATP. With respect to adenine nucleotide-mediated aggregation in platelets, the ADP-activated P2Y12 receptor is the most important receptor. P2Y12 was first discovered in 2001 (Hollopeter et al., 2001), and it can couple to the G_αi_ subunit leading to the inhibition of adenylyl cyclase that mediates the conversion of ATP to cAMP (Hollopeter et al., 2001; Cattaneo, 2007; Hechler and Gachet, 2011). Consequently, the overall levels of cAMP can be decreased which may lead to an increase in platelet activation state. One of the mechanisms by which cAMP reduces platelet aggregation involves activation of protein kinase A (PKA) which leads to the phosphorylation and inhibition of inositol 1,4,5-triphosphate (IP3) receptor-mediated increase of calcium from the dense tubular system; reduced levels of cAMP have the opposite effect on IP3 and consequently increase intracellular calcium (Quinton and Dean, 1992; Tertyshnikova and Fein, 1998). In addition to coupling through G_αi_ and inhibition of adenylyl cyclase, ADP-mediated activation of P2Y12 receptors leads to activation of PI-3 kinase (PI-3K) through G_βγ_ (Shankar et al., 2004). It is now understood that the P2Y12 receptor contributes to the ADP-mediated calcium response (by P2Y1) in platelets by activating PI-3K, in addition to simultaneously inhibiting adenylyl cyclase, i.e., by reducing cAMP levels (Hardy et al., 2004). Importantly, ADP activation through G_αi_-coupled P2Y12 receptors and the consequent RAP1 activation is critical for GPIIb/IIIa integrin-mediated platelet aggregation (Larson et al., 2003; Stefanini and Bergmeier, 2017). The P2Y12, but not P2Y1 receptor is required for sustained activation of RAP1 (Ras-related protein 1). P2Y12 is necessary for the formation of a shear-resistant hemostatic plug which is mediated by locally secreted ADP from platelets (Larson et al., 2003; Stolla et al., 2011b; Stefanini and Bergmeier, 2017). RAP1 activation is also dependent on guanine exchange factor (GEF), proteins RapGEF and RAS guanyl releasing protein 2 (RASGRP2), that are regulated by intracellular calcium signaling and by inhibition from the GTP-ase activating protein Ras GTPase-activating protein 3 (RASA3) (Stolla et al., 2011a; Stefanini and Bergmeier, 2017). ADP-activated P2Y1 receptor, on the other hand, does not affect cAMP levels (Fabre et al., 1999), couples to the G_αq_ subunit leading to the activation of phospholipase C (PLC) (Cattaneo, 2007; Hechler and Gachet, 2011). In platelets, PLC activation leads to a cascade of molecular signaling events that result in increased levels of cytosolic calcium, particularly from the dense tubular system. Interestingly, the P2Y1 receptor is able to activate Src kinase and consequently establish negative regulation over P2Y12 signaling through PI-3K (Hardy et al., 2004). This suggests that for ADP-mediated calcium signaling in platelets, P2Y12 and P2Y1 receptors cross-communicate to establish a controlled response to vascular damage; the P2Y12 receptor contributes to the absolutely required P2Y1-mediated calcium response by activating PI-3K and reducing cAMP levels, while the P2Y1 receptor inhibits PI-3K by coupling with Src kinase (Hardy et al., 2004). Thus, now it is understood that, in addition to ADP activation of platelets through two different molecular signaling mechanisms in which platelet activation by P2Y1 leads to rapid shape change and reversible aggregation while P2Y12 activation induces sustained platelet aggregation but without a shape change (Cattaneo, 2007; Hechler and Gachet, 2011), there is also a synergy between these receptors at the level of calcium signaling (Hardy et al., 2004). Importantly, other platelet G-protein-coupled receptor signaling through G_αq_ and contribution to the overall calcium pool can further synergize with ADP receptors and affect platelet function. Detailed reviews of platelet P2 signaling and synergy at calcium regulation can be found elsewhere (Dorsam and Kunapuli, 2004; Cattaneo, 2007; Varga-Szabo et al., 2009; Hechler and Gachet, 2011; Stefanini and Bergmeier, 2017).

It is important to mention that the role of ADP secreted from platelet dense-granules is to amplify the aggregation signal induced by other strong platelet agonists (such as TxA2), assuring stable platelet aggregation. Conversely, coordinated signaling involving activation of the P2Y12 and P2Y1 ADP receptors and integrin GPIIb/IIIa can mediate TxA2 generation (Jin et al., 2002). This multifactorial ADP receptors–TxA2 axis in platelets is important for thrombus growth and stabilization. Interestingly, patients deficient only in P2Y12 receptors have unaffected TxA2 production (Cattaneo et al., 1992, 2000), suggesting an important requirement of the two ADP receptors for proper hemostasis.

Studies utilizing murine platelets deficient in P2Y1 receptor showed that these platelets lack shape change and are prone to decreased aggregation in response to high concentrations of ADP (Fabre et al., 1999; Leon et al., 1999). However, P2Y1-deficient platelets had strongly impaired aggregation at low concentrations of ADP or collagen (Leon et al., 1999), suggesting that the P2Y1 receptor contributes to the overall ADP-mediated platelet aggregation and responses to other agonists. On the other hand, platelet overexpression of the P2Y1 receptor induces platelet hyperactivity (Hechler et al., 2003b). Bleeding time of mice deficient in this receptor is increased and they are protected from collagen- or ADP-induced thromboembolism (Fabre et al., 1999; Leon et al., 1999), suggesting that P2Y1 mediates ADP–platelet aggregation effects *in vivo*. Studies utilizing a murine model deficient in P2Y12 (Andre et al., 2003) in the context of the mesenteric artery injury model, support the observation that this receptor mediates platelet adhesion and activation, in addition to mediating the growth and stability of the thrombus. Consistently, *in vivo* antagonism of the P2Y12 receptor in rabbits reduces the size of the initial thrombus in injured mesenteric arterioles. However, in this study utilizing P2Y12 antagonists, AR-C69931 MX or clopidogrel (vs. genetic elimination), the stability of the thrombus was not affected by P2Y12 and the formation of the hemostatic plug was also not affected (van Gestel et al., 2003).

There are various reasons as to why a genetic deletion may lead to a different outcome than pharmacological inhibition in growth and stability of the thrombus. An inhibitor can vary in binding affinity for the receptor, reversibility, availability at specific sites, and off-target effects, as compared to a complete lack of receptor expression. Genetic deletion, on the other hand, can lead to elimination not only of the gene of interest but can also alter levels of other genes that may be transcriptionally regulated by the particular gene. Finally, since human and murine platelets exhibit differences in content, the differences in pharmacological inhibition vs. deletion can additionally be related to species variability. Regardless, although an antagonist may not completely inhibit the action of P2Y12 or account for other functions of P2 receptors, it is clear that inhibition of P2Y12 can be beneficial in limiting thrombus growth in arterial thrombosis and secondary prevention of ischemia (Andre et al., 2003; van Gestel et al., 2003).

## Platelets and P1 Receptors

### Adenosine Receptors in Platelets

In addition to P2 receptors, platelets express functional P1 purinergic receptors on their surfaces. As mentioned earlier, P1 purinergic receptors are activated by adenosine at various concentrations that may be generated by ATP/ADP hydrolysis. In addition, an increase in levels of nucleotides, and consequent adenosine generation, can occur during inflammation, cell damage during infection, or other cardiovascular pathologies (Fredholm, 2007; Koupenova and Ravid, 2013). Human and murine platelets express functional cAMP-increasing P1 receptors A2aAR and A2bAR (Ledent et al., 1997; Yang et al., 2010), but are not known to have functional cAMP-inhibiting P1 receptors A1AR and A3AR. It is noteworthy that A1AR mRNA has been detected by qRT-PCR (Amisten et al., 2008) but significant expression was not detected by sequencing in more recent studies (Rowley et al., 2011; Clancy et al., 2017). An increased level of intracellular cAMP, in turn, inhibits platelet aggregation/function.

Among the A2 adenosine receptors, A2aAR has the highest affinity for adenosine and is the major adenosine receptor expressed in platelets that mediates inhibition of platelet aggregation. A2aAR is a G-protein-coupled receptor that couples with G_αs_ and activates adenylyl cyclase (Koupenova et al., 2012). A2aAR mediates platelet function through increasing cAMP levels (Yang et al., 2010), and by inhibiting the rise of thrombin-mediated increase in intracellular calcium levels (Paul et al., 1990). Both of these effects on intracellular second messengers in platelets are presumed to be mediated by the action of adenylyl cyclase (Paul et al., 1990). It is now understood that the inhibitory effect of cAMP on platelet aggregation involves complex regulation of diverging signaling pathways. Intracellularly, cAMP affects calcium signaling by inhibiting PLC-mediated diacylglycerol (DAG) and inositol triphosphate formation, by inhibiting protein kinase C directly or indirectly through reduction of DAG level, or by affecting actin polymerization through phosphorylation of the β-subunit of the glycoprotein receptor GPIb (Fox and Berndt, 1989; Schwarz et al., 2001; Varga-Szabo et al., 2009). In addition to inhibiting platelet aggregation in human blood, activation of A2aAR by specific agonists leads to a reduction in P-selectin expression on the platelet cell surface, as a result of TxA2 or ADP stimulation. This is followed by a greater than 50% reduction in platelet–monocyte aggregate formation (Linden et al., 2008). This suggests that the A2aAR has the potential to mediate the impact of prothrombotic agonists during hemostasis and thrombosis as a fast responder.

Studies utilizing platelets from A2aAR null mice have confirmed that inhibition of platelet aggregation is mediated by this receptor, as platelets without A2aAR have an increased aggregation potential and are not affected by any concentration of the adenosine receptor (non-specific) agonist, 5′-*N*-ethylcarboxamidoadenosine (NECA) (Ledent et al., 1997). Similarly, inhibition of A2aAR by caffeine, a non-specific adenosine receptor antagonist, also leads to decreased aggregation potential of human platelets (Varani et al., 1999, 2000). However, chronic intake of caffeine leads to a desensitization effect on aggregation, possibly mediated by increased upregulation of A2aAR (or A2bAR) on the platelet surface (Varani et al., 1999, 2000).

Although platelets have a large density of A2aAR, they also contain A2bARs. Similarly to A2aAR, A2bAR is a G-protein receptor that couples to G_αs_ (Koupenova et al., 2012), but can also couple to G_αq_ (Feoktistov et al., 1994; Linden et al., 1999; Gao et al., 2017) and, in some cells, to G_αi_ (Gao et al., 2017). Importantly, in different cells, A2bAR can couple to the same pathways through different G-proteins possibly depending on specific intracellular G-protein signatures (Gao et al., 2017). In cells of the same type, A2bAR can also couple to G_αs_ or G_αi_ leading to activation of opposing or synergistic signaling pathways (Gao et al., 2017), suggesting that these adenosine receptors have the ability to balance different signaling mechanisms in different cell types. Contrary to the other P1 receptors, A2bAR is a low affinity receptor that requires the concentration of adenosine to be higher than 10 μM (reviewed in Koupenova and Ravid, 2013). Additionally, A2bAR is an inducible receptor, the expression of which is known to be upregulated under inflammation, stress, or vascular injury (Yang et al., 2006, 2008, 2010; St. Hilaire et al., 2008). Since platelets are anucleate cells, increase in A2bAR expression most likely occurs in the platelet precursors, the megakaryocytes (MK). The low affinity for adenosine, the inducibility of A2bAR expression, and the promiscuous coupling with downstream G-proteins suggest that this receptor may play an instrumental role in platelet function during chronic inflammation and pathophysiological conditions.

Recent sequencing studies show that mRNA expression of A2bAR in human platelets is almost sixfold lower than A2aAR (**Table 1**) (Rowley et al., 2011). Murine platelets lacking A2bAR have reduced cAMP levels and are prone to increased ADP- or collagen-mediated aggregation (Yang et al., 2010). Additionally, A2bAR increases the inhibition of ADP-mediated aggregation of platelets isolated from LPS-injected animals (Yang et al., 2010). Elimination of A2bAR also leads to increased platelet expression of the P2Y1 receptor (Yang et al., 2010), and P2Y1 activation, as mentioned before, leads to an increase in intracellular calcium levels (Cattaneo, 2007; Hechler and Gachet, 2011). This suggests that the absence of A2bAR can have a stimulatory effect on platelet aggregation through simultaneous reduction of cAMP and increase in calcium levels. A2bAR signaling could be particularly important over prolonged periods of inflammation or injury.

**Table 1:** Platelet purinergic receptors assessed by RNA sequencing of healthy human or murine platelets.

Purinergic receptor	Nucleotide/nucleoside	G-Protein coupling	Effect on platelet cAMP	Effect on platelet calcium	*Large human* platelets (Clancy et al., 2017) (FPKM)	*Small human* platelets (Clancy et al., 2017) (FPKM)	*All human* platelets (Rowley et al., 2011) (RPKM)	*All murine* platelets (Rowley et al., 2011) (RPKM)
**P1 Purinergic receptors**								
A1AR	Ado	G_αi_	–	–	0	0	0.05	0
A3AR	Ado	G_αi_	–	–	0	1.80	0.08	1.86
A2aAR	Ado	G_αs_	↑	–	0	2.23	31.80	0.36
A2bAR	Ado	G_αs_	↑	–	0	0	5.37	0
**P2 Purinergic receptors**								
P2X1	ATP	Ion-gated channel	–	↑	4.21	1.55	90.79	630.06
P2Y1	ADP	G_αq_	–	↑	0	0	12.53	30.56
P2Y12	ADP	G_αi_	↓	–	13.19	1.77	73.75	1044.24

*The two studies summarized here included healthy human donors and isolated platelets by sorting [large and small platelets (Clancy et al., 2017)] or by centrifugation followed by leukocyte depletion (Rowley et al., 2011). The human cohorts, methods of platelet isolation, and RNA extractions are described in Rowley et al. (2011) and Clancy et al. (2017). Mice sequencing data represent RNA transcripts in platelets from male and female C57BL/6 murine models (Rowley et al., 2011). Of note: The data summarized here are derived from platelets from two human male donors (large and small platelets) and from one male and one female donor (data in “all platelet” column). Data validation is required in various individuals of different ages and sexes in order to establish a broader distribution profile of purinergic receptors. cAMP, cyclic AMP; FPKM, fragments per kilobase of transcript per million mapped reads; RPKM, reads per kilobase of transcript per million mapped reads; Ado, Adenosine; ATP, adenosine triphosphate; ADP, adenosine diphosphate.*

## Purinergic Receptors and Platelet Heterogeneity

Circulating platelets are anucleate cell fragments originating from their bone marrow precursor, the MK. Human platelets are not a homogeneous population, but vary in size (2–5 μm) and content. RNA sequencing shows that platelets may contain as many as 9500 different transcripts (Rowley et al., 2011; Bray et al., 2013; Clancy et al., 2017), most of which are prepackaged from the MK. Additionally, platelets uptake transcripts and transcript fragments from circulating cells and the endothelium (Clancy et al., 2017). The heterogeneous size of platelets (Thon and Italiano, 2012) is hypothesized to be a result of platelets losing their contents into the circulation as they age; hence, large platelets are also referred to as “immature” and small platelets are referred to as “mature” (Penington and Streatfield, 1975; Penington et al., 1976a,b). Alternatively, in the late 1970s, it was proposed that platelets vary in density and structure depending on ploidy of the MK from which platelets are originating. Low ploidy MKs produce less hemostatically active platelets while high ploidy MKs produce hemostatically functional platelets (Penington and Streatfield, 1975; Penington et al., 1976a,b). Direct experimental evidence is limited for each of these hypotheses and it is possible that both of these explanations are valid. Studies have shown that large (immature) platelets are highly hemostatically active while small (mature) platelets are less hemostatically functional (Thompson et al., 1984; Guthikonda et al., 2008). It is noteworthy that the predominant size of platelets in a healthy individual lies somewhere in between (Clancy et al., 2017). In patients with acute coronary syndrome (developing early stent thrombosis) there is a baseline platelet size increase predicting platelet reactivity (Huczek et al., 2010). A population study performed with patients after non-cardiac surgery has provided evidence that large platelets may be a novel biomarker for adverse cardiovascular events (Anetsberger et al., 2017).

**FIGURE 1:**
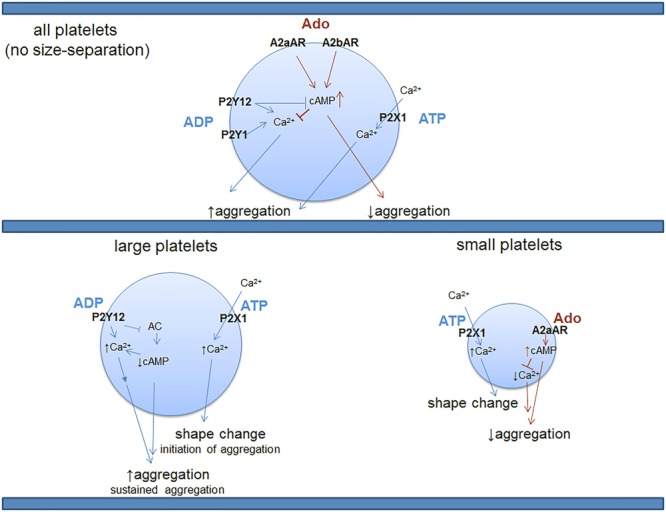
Distribution of purinergic receptor transcripts with known function in the entire platelet population vs. large and small platelets. Platelets are a heterogeneous population of various sizes. Large platelets are highly hemostatically active while small ones are known to be much less active in hemostasis (Thompson et al., 1984; Guthikonda et al., 2008). RNA sequencing studies shown in **Table 1** suggest differential distribution of purinergic receptors across platelets of different sizes, providing a provocative hypothesis related to potential functional differences in hemostasis when activated by adenine nucleosides and nucleotides, depending on platelet size. Future protein studies will be needed to test this contention. P2X1 is a ligand-gated ion channel that requires binding of ATP for influx of calcium; the rest of the P2 and P1 receptors in platelets are G-protein-coupled receptors. Of note, with the sensitivity of the above method of detection, P2Y1 or A2bAR transcripts are not found in the small or large platelets; however, these receptors for ADP or adenosine (respectively) are detected in the entire platelet population, suggesting a differential expression level in different platelet populations, possibly necessary for an extra layer of control over platelet function. These findings further encourage future examination of the receptors at protein and functional levels in different platelet populations. It is not known, however, if all of the purinergic receptors depicted in the top (or bottom) panel can be expressed on the same platelet, if there is differential signature of co-expression or if there is a mix of both of these possibilities. Ado, adenosine; ATP, adenosine triphosphate; ADP, adenosine diphosphate; cAMP, cyclic adenosine monophosphate; AC, adenylate cyclase.

Sequencing studies have addressed the overall platelet mRNA transcriptome of the entire platelet population (Rowley et al., 2011) and that of small and large platelets sorted from the blood of healthy human donors (Clancy et al., 2017). Of note, in platelets there is a reported mismatch between mRNA and protein expression (Burkhart et al., 2012) that can be partially justified by the ability of platelets to uptake mRNA transcripts (Clancy et al., 2017). However, in cases when both proteins and transcripts are detectable in platelets, there is strong evidence that mRNA and the corresponding protein expression are correlated (Rowley and Weyrich, 2013). Large platelets (sorted as 10% of the entire platelet population) have transcripts associated with classical platelet functions, such as platelet activation/aggregation, hemostasis, and wound healing (Clancy et al., 2017). Small platelets show a distinct and more diverse platelet transcriptome as compared to large platelets, and those transcripts are more involved in platelet-immune cell interactions and apoptosis (Clancy et al., 2017). For instance, small platelets contain distinct transcripts that are associated with activation, proliferation and differentiation of T- and B-lymphocytes (Clancy et al., 2017). Consistent with the hemostatic role of large platelets and their contribution to aggregation, transcripts for the P2 purinergic receptors, P2Y12 and P2X1, seem to be present at higher levels in this platelet population (**Table 1**). mRNA from the inhibitory P1 receptor A2aAR is distinctly located in small platelets, similar to P1 A3AR (**Table 1**). The function of platelet-A3AR is unclear. On the other hand, sequencing of the entire platelet population, that includes platelets of all sizes, shows the presence of all four P1 purinergic receptors with the inhibitory A2aAR and A2bAR receptors in the highest proportions (**Table 1**). Interestingly, P2Y1 is detected only in the mixed, non-sorted platelet population, but not in the large or small platelet subpopulations. There is a possibility that the P2Y1 receptor is present on platelets which compose the rest of the platelet population that was not included in the large/small platelet sorting, or P2Y1 was not detected due to low expression levels in each group or in these particular donors. The two sequencing studies also agree on transcripts for other P2 purinergic receptors such as P2X5, P2Y10, and P2Y13 that have not been previously reported to be present in platelets or to have a functional role. Distinct signatures of purinergic receptors in different platelet subpopulations suggests that the architecture of the hemostatic plug may involve platelets of heterogeneous size and function. Large platelets may be predominantly located at the core while small platelets may be located (together with the large) in the shell (**Figure 1**). Since small platelets have more immune transcripts and are less hemostatically active, their presence may allow for direct interactions with immune cells inside of the shell of the thrombus. The mechanism by which MKs mediate this diverse distribution of distinct transcripts in platelets according to size and function is still controversial.

## Conclusion and Future Directions

The distinct distribution of purinergic receptor types across different platelet sizes may provide a new approach to purinergic signaling manipulation in order to establish effective antithrombotic therapies. The platelet mRNA profile may have a broader impact on overall platelet function, and proposes an explanation of previously identified functional differences between small and large platelets, with increased mean platelet volume being historically associated with increased hemostatic potential. It remains unclear, however, if within the sophisticated architecture of the thrombus there is a distinct arrangement of platelets with differential expression of purinergic or immune receptors. Perhaps, big platelets are primarily responsible for the formation of the core of the hemostatic plug since they predominantly contain transcripts related to the hemostatic function of platelets, such as the P2 receptors. Small platelets in turn contain more immune transcripts. These cells may need to communicate with both aggregated platelets and leukocytes and may be located in the porous shell of the hemostatic plaque. In that sense, it would be necessary for small platelets to be less prone to aggregation and hence express only purinergic receptors for shape change (P2X1) and reduction of aggregation (A2ARs). It is also not completely clear if platelet heterogeneity increases as a function of pathological conditions, thereby changing the balance between P2/P1 receptors and limiting the beneficial effect of current therapies.

It would be important to test these receptors at protein and agonist functional levels in the different platelet subpopulations. Additionally, future studies are necessary to elucidate the complex cross-communication of purinergic receptors in the hemostatic plug as a function of platelet heterogeneity and to determine if there is a functional presence of the other ADP/ATP receptors that were detected at mRNA level. These future findings may provide additional pharmacological targets in the management of thrombus growth and stability in patients.

## Author Contributions

MK and KR co-wrote and edited this review article.

## Conflict of Interest Statement

The authors declare that the research was conducted in the absence of any commercial or financial relationships that could be construed as a potential conflict of interest.
